# Supplementary material for: Modeling glioblastoma heterogeneity as a dynamic network of cell states
Source: Mol Syst Biol. 2021 Sep 16;17(9):e10105. doi: 10.15252/msb.202010105 (PMC8444284; doi:10.15252/msb.202010105)
Supplement: Supplementary file 5 — Source Data for Figure 3 [file MSB-17-e10105-s001.zip › Figure3A_sourcedata/GSEA_3065/nosigdb_state3.GseaPreranked.1622718426028/IVY_CTMVP.html]

Details for gene set IVY\_CTMVP[GSEA]

|  || Dataset | state3 |
| Phenotype | NoPhenotypeAvailable |
| Upregulated in class | na\_pos |
| GeneSet | IVY\_CTMVP |
| Enrichment Score (ES) | 0.5639341 |
| Normalized Enrichment Score (NES) | 1.8670459 |
| Nominal p-value | 0.002631579 |
| FDR q-value | 0.002892036 |
| FWER p-Value | 0.03 |
Table: GSEA Results Summary

  

Fig 1: Enrichment plot: IVY\_CTMVP      
 Profile of the Running ES Score & Positions of GeneSet Members on the Rank Ordered List

  

| PROBE | GENE SYMBOL | GENE\_TITLE | RANK IN GENE LIST | RANK METRIC SCORE | RUNNING ES | CORE ENRICHMENT || 1 | LAMC1 |  |  | 96 | 0.349 | 0.0480 | Yes |
| 2 | ITGB1 |  |  | 150 | 0.310 | 0.0942 | Yes |
| 3 | COL4A2 |  |  | 232 | 0.271 | 0.1307 | Yes |
| 4 | COL4A1 |  |  | 241 | 0.267 | 0.1748 | Yes |
| 5 | CTGF |  |  | 263 | 0.257 | 0.2157 | Yes |
| 6 | COL5A2 |  |  | 266 | 0.255 | 0.2585 | Yes |
| 7 | MCAM |  |  | 356 | 0.229 | 0.2870 | Yes |
| 8 | PXDN |  |  | 362 | 0.227 | 0.3248 | Yes |
| 9 | LAMB1 |  |  | 415 | 0.215 | 0.3552 | Yes |
| 10 | LAMA4 |  |  | 496 | 0.199 | 0.3796 | Yes |
| 11 | PCOLCE |  |  | 533 | 0.194 | 0.4083 | Yes |
| 12 | A2M |  |  | 585 | 0.188 | 0.4342 | Yes |
| 13 | SERPINH1 |  |  | 588 | 0.188 | 0.4657 | Yes |
| 14 | COL18A1 |  |  | 598 | 0.186 | 0.4962 | Yes |
| 15 | FSTL1 |  |  | 790 | 0.159 | 0.5010 | Yes |
| 16 | TM4SF1 |  |  | 952 | 0.142 | 0.5066 | Yes |
| 17 | FN1 |  |  | 973 | 0.140 | 0.5280 | Yes |
| 18 | GPX8 |  |  | 1138 | 0.127 | 0.5306 | Yes |
| 19 | OLFML2A |  |  | 1150 | 0.126 | 0.5506 | Yes |
| 20 | F2R |  |  | 1308 | 0.115 | 0.5520 | Yes |
| 21 | NID1 |  |  | 1545 | 0.101 | 0.5419 | Yes |
| 22 | THBS1 |  |  | 1561 | 0.100 | 0.5571 | Yes |
| 23 | KDELR3 |  |  | 1678 | 0.093 | 0.5595 | Yes |
| 24 | STEAP1B |  |  | 1772 | 0.088 | 0.5636 | Yes |
| 25 | PRSS23 |  |  | 1890 | 0.081 | 0.5639 | Yes |
| 26 | TGFBI |  |  | 2753 | 0.049 | 0.4732 | No |
| 27 | UACA |  |  | 2971 | 0.042 | 0.4553 | No |
| 28 | KCNJ2 |  |  | 2991 | 0.041 | 0.4600 | No |
| 29 | TGFBR2 |  |  | 3803 | 0.020 | 0.3703 | No |
| 30 | FRZB |  |  | 5464 | -0.023 | 0.1835 | No |
| 31 | ARHGAP18 |  |  | 5579 | -0.027 | 0.1749 | No |
| 32 | IGFBP7 |  |  | 5667 | -0.029 | 0.1698 | No |
| 33 | TPM2 |  |  | 5823 | -0.034 | 0.1577 | No |
| 34 | STC1 |  |  | 5905 | -0.036 | 0.1544 | No |
| 35 | SVIL |  |  | 6089 | -0.042 | 0.1405 | No |
| 36 | CCDC102B |  |  | 6213 | -0.047 | 0.1343 | No |
| 37 | ARHGAP29 |  |  | 6596 | -0.060 | 0.1007 | No |
| 38 | LBH |  |  | 6651 | -0.064 | 0.1052 | No |
| 39 | TAGLN |  |  | 6722 | -0.067 | 0.1085 | No |
| 40 | PRDM1 |  |  | 6839 | -0.072 | 0.1074 | No |
| 41 | FILIP1L |  |  | 6892 | -0.075 | 0.1142 | No |
| 42 | PDLIM1 |  |  | 6965 | -0.079 | 0.1192 | No |
| 43 | PHACTR2 |  |  | 7551 | -0.122 | 0.0727 | No |
| 44 | GNG11 |  |  | 8577 | -0.385 | 0.0200 | No |
Table: GSEA details [plain text format]

  

Fig 2: IVY\_CTMVP: Random ES distribution      
 Gene set null distribution of ES for **IVY\_CTMVP**
